# Antiferromagnetic Ordering of Magnetic Clusters Units in Nb_6_F_15_

**DOI:** 10.1007/s00723-012-0426-6

**Published:** 2012-12-15

**Authors:** R. Knoll, A. Shames, S. D. Goren, H. Shaked, S. Cordier, C. Perrin, O. Hernandez, T. Roisnel, G. André, R. K. Kremer, A. Simon

**Affiliations:** 1 Department of Physics, Ben Gurion University, P.O. Box 653, 84105 Beer Sheva, Israel; 2 Sciences Chimiques de Rennes, UMR 6226 CNRS, Université de Rennes 1, Avenue du Général Leclerc, Bât. 10A/10B, 35042 Rennes Cedex, France; 3 CEA/Saclay, 91191 Gif-sur-Yvette Cedex, France; 4 Max-Planck-Inst. f. Festkörperforschung, Heisenbergst. 1, 7000 Stuttgart 80, Germany

## Abstract

We have studied the magnetic cluster compound Nb_6_F_15_ which has an odd number of 15 valence electrons per (Nb_6_F_12_)^3+^ cluster core, as a function of temperature using nuclear magnetic resonance, magnetic susceptibility, electron magnetic resonance and neutron powder diffraction. Nuclear magnetic resonance of the ^19^F nuclei shows two lines corresponding to the apical F^a−a^ nucleus, and to the inner F^i^ nuclei. The temperature dependence of the signal from the F^i^ nuclei reveals an antiferromagnetic ordering at *T* < 5 K, with a hyperfine field of ~2 mT. Magnetic susceptibility exhibits a Curie–Weiss behavior with *T*_*N*_ ~5 K, and *μ*_eff_ ~1.57 μ_B_ close to the expected theoretical value for one unpaired electron (1.73 μ_B_). Electron magnetic resonance linewidth shows a transition at 5 K. Upon cooling from 10 to 1.4 K, the neutron diffraction shows a decrease in the intensity of the low-angle diffuse scattering below *Q* ~0.27 Å^−1^. This decrease is consistent with emergence of magnetic order of large magnetic objects (clusters). This study shows that Nb_6_F_15_ is paramagnetic at RT and undergoes a transition to antiferromagnetic order at 5 K. This unique antiferromagnetic ordering results from the interaction between magnetic spins delocalized over each entire (Nb_6_F_12_^i^)^3+^ cluster core, rather than the common magnetic ordering.

## Introduction

**Fig. 1:**
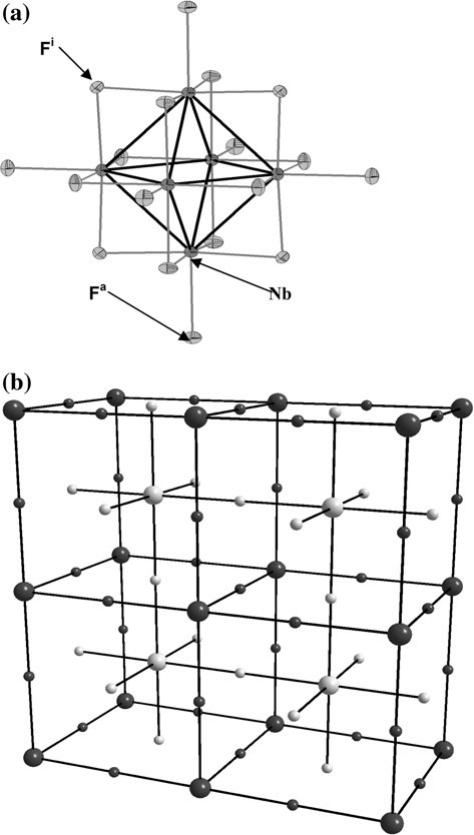
**a** Nb_6_F_12_^i^F_6_^a^ cluster unit. **b** Schematic representation of the interconnection of units in Nb_6_F_15_. The *large spheres* represent the (Nb_6_F_12_^i^)^3+^ cluster core and the *small ones* represent the apical (F^a−a^)^−^ ligands bridging the cluster cores

Metallic octahedral clusters are characterized by metal–metal bond lengths close to those found in the corresponding metals. Indeed, the Nb–Nb bond length in niobium metal is found to be 2.858 Å [1] compared with 2.794 Å in Nb_6_F_15_ [2]. Cluster compounds are easily obtained, via solid-state synthesis at high temperature, with early transition metal elements in their low oxidation state. The Nb_6_L_18_ unit (L = O, F, Cl, Br) [4–6] constitutes the basic building block in the octahedral niobium cluster chemistry. The Nb_6_ octahedron is surrounded by 12 L^i^ ligands (i = inner) in edge bridging positions, and by six L^a^ ligands (a = apical) in terminal positions (Fig. 1a). This discrete unit is then written Nb_6_L_12_^i^L_6_^a^, according to the Schäfer notation [7]. Theoretical calculations of the M_6_L_18_ unit electronic structure, considering an *O*_*h*_ symmetry, show a set of four molecular orbitals (*t*_2*g*_, *a*_1*g*_, *t*_1*u*_, *a*_2*u*_)—two of them being triply degenerated—that can accommodate 16 electrons. These molecular orbitals show a strong Nb–Nb bonding character. Their full occupation leads to a closed-shell configuration with 16 electrons per Nb_6_ cluster [8].

The use of fluorine as ligand enables to stabilize original structures in which the edge-bridged cluster units are interconnected through linear Nb–F^a−a^–Nb bridges [9–12]. This class of solid-state compounds constitutes a very rare example of fluorides containing metal–metal bonds. The association of magnetic clusters with 15e^−^/Nb_6_ with fluorine ligands must lead to molecular compounds in which magnetic interactions should occur as already found for many other fluorides [13]. Despite the fact that the binary Nb_6_F_15_ has been characterized a long time ago, its physical properties have not been fully investigated. It is built up from [Nb_6_L_12_^i^L_6_^a^] cluster units interconnected by sharing the six F^a^ ligands in the three directions (Fig. 1b). Each F^a^ is shared by two (Nb_6_F_12_^i^)^3+^ cluster cores and will be noted as F^a−a^. The formation of the linear Nb–F^a−a^–Nb bridges along the axes of the cubic unit-cell leads to a Nb_6_F_12_^i^F_6/2_^a−a^ cluster framework.

The (Nb_6_F_12_^i^)^3+^ cluster core is characterized by an odd number of 15 valence electrons per cluster (VEC), with the unpaired electron occupying the a_2u_ Nb–Nb bonding highest occupied molecular orbital (HOMO), leading to a magnetic behavior [14]. The room temperature crystal structure has been very recently re-refined through single-crystal X-ray diffraction [2] to yield more precise lattice, atomic and geometric parameters. The space group is *Im*-3 *m*, *a* = 8.1878(2) Å, *Z* = 2. Owing to the body-centered lattice, the structure can also be described by two interpenetrating simple cubic (SC) lattices of cluster units related to each other by a (½ ½ ½) translation (Fig. 1b). Recently, ^19^F nuclear magnetic resonance (NMR) experiments [2a] have been performed on powder sample. The signal exhibits two resonance lines, one attributed to the F^i^ nuclei, the other one to the F^a−a^ nuclei. From 300 down to 75 K, only the former line displays temperature dependence (in this instance of Curie-like type). This result follows from the fact that the F^i^ low-symmetry site is sensitive to the magnetism of the cluster, whereas the F^a−a^ is located on a site with a high (cubic) symmetry, resulting in the cancelation of the magnetic contribution from neighboring clusters. Electron magnetic resonance (EMR) experiments [2] down to *T* = 4 K confirm the Curie–Weiss magnetic behavior and indicate the possibility of magnetic ordering of the cluster units below *T*_*N*_ ~5 K. SQUID measurements of LuNb_6_Cl_18_, another Nb_6_ cluster compound with a VEC value of 15, also indicate such a transition [15, 16].

In the present work, we investigate by the means of different experimental techniques (NMR, magnetic susceptibility, EMR and neutron powder diffraction) the low-temperature magnetic behavior of this binary cluster fluoride.

## Experimental Procedure

The preparation of Nb_6_F_15_ was outlined in an earlier publication [2, 3].

### NMR

The NMR data were measured in an external field of 0.7 T with a Tecmag NMR spectrometer over a temperature range of 3.3–75 K with a 16-pulse sequence [17]. The length of the 90º pulse was 1 μs and a repetition rate of 20 ms was used for studying the spectra originating from the inner F^i^ against a repetition rate of 2 s for the data originating from the apical F^a−a^.

### Magnetic Susceptibility

Magnetic susceptibility data were measured over a temperature range of 2–300 K in an external magnetic field of 0.2 T in order to suppress the diamagnetic signal originating from Nb metal impurities.

### EMR

EMR spectra were measured with a Bruker EMX spectrometer operating at X-band (9.4 GHz) over a temperature range of 3–50 K.

### Neutron Powder Diffraction

Neutron powder diffraction data were collected using the 800 cells curved multi-detector at the cold-neutron two-axis spectrometer G41, at the Orphée reactor, Laboratoire Leon Brillouin, Saclay. A vertically focusing graphite monochromator ( *λ* = 2.423 Å), followed by a graphite filter, produced a 50′10 mm^2^ neutron beam at the sample position. The as prepared Nb_6_F_15_ powder was loaded into an 8-mm vanadium sample holder which was mounted in a cryostat that was placed at the sample position. Neutron data were collected for 4° ≤ 2θ ≤ 84° (i.e. 0.18 Å^−1^ ≤ *Q* ≤ 3.47 Å^−1^ in reciprocal space) at 10 K and 1.4 K (25 h of counting time at each temperature).

## Experimental Results

### NMR

**Fig. 2:**
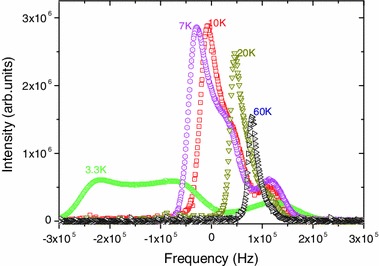
^19^F NMR spectra of Nb_6_F_15_ at various temperatures. The F^a−a^ line is hardly visible at 60 K

Results of our previous work [2] as well as the present work show that at *T* > 75 K the ^19^F NMR spectra of Nb_6_F_15_ consist of 2 lines, the high-frequency symmetric line corresponding to the apical F^a−a^ and the low-frequency asymmetric line to the inner F^i^. The position of the F^i^ line showed a Curie–Weiss temperature dependence whilst the position of the F^a−a^ line is temperature-independent. Figure 2 gives (present work) the low-temperature behavior of the ^19^F NMR spectra of Nb_6_F_15_. The NMR line of the inner F^i^ shows a marked change in the lineshape for *T* = 3.3 K. At this temperature, this line has a double hump lineshape, as expected for polycrystalline antiferromagnetic material [18].

**Fig. 3:**
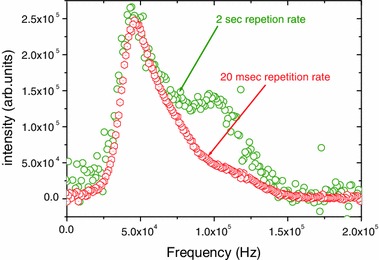
^19^F NMR spectra of Nb_6_F_15_ at *T* = 20 K. The *lower trace* gives the results for a repetition rate of 20 ms while the *upper trace* shows the results for a repetition rate of 2 s

Figure 3 shows the signal obtained with a repetition rate of 20 ms (same repletion rate as in Fig. 2) and a repetition rate of 2 s. While the signal from the inner F^i^ hardly changes, there is a marked increase in the intensity of the signal from the apical F^a−a^. This effect was discussed in a previous work [2, 3]. This repetition rate was used to obtain the position of the signal of the apical F^a−a^.

**Fig. 4:**
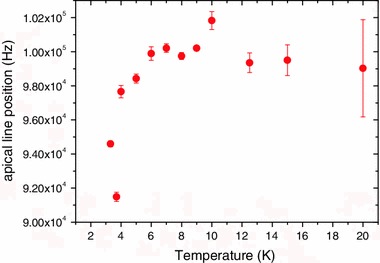
Position of the apical F^a−an^ NMR line as a function of the temperature

Figure 4 shows the temperature dependence of the position of NMR line of the apical F^a−a^. For *T* > 6 K, the position of this line is temperature-independent, while for *T* < 6 K the position depends on the temperature. This temperature behavior of the NMR line of the apical F^a−a^ has to be compared with the temperature behavior of the NMR line of the inner F^i^ which shows a Curie–Weiss temperature dependence [2].

### Magnetic Susceptibility Measurements

**Fig. 5:**
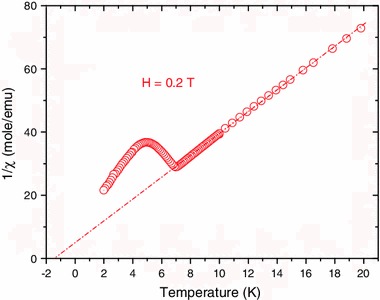
Reciprocal magnetic susceptibility of Nb_6_F_15_ at *H* = 0.2 T as a function of the temperature

Figure 5 shows the reciprocal magnetic susceptibility of Nb_6_F_15_ as a function of the temperature measured in an external magnetic field of 0.2 T. The onset of the magnetic transition is at *T* ~7 K (while the transition occurs at *T*_*N*_ = 5 K). The measured effective magnetic moment deduced from this curve is 1.57 µ_B_, which is close to the value of √3 µ_B_ of a single electron per cluster.

### EMR

**Fig. 6:**
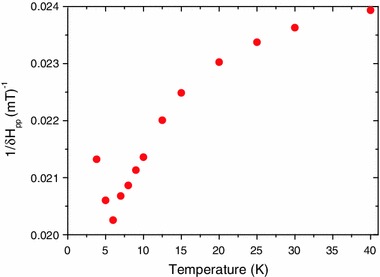
Reciprocal linewidth of the EMR spectra as a function of the temperature

Figure 6 gives the low-temperature dependence of the reciprocal linewidth of the EMR line. The onset of the transition is at *T* ~7 K. Note that this graph is a qualitative replication of the graph of the reciprocal magnetic susceptibility (Fig. 5).

### Neutron Powder Diffraction

**Fig. 7:**
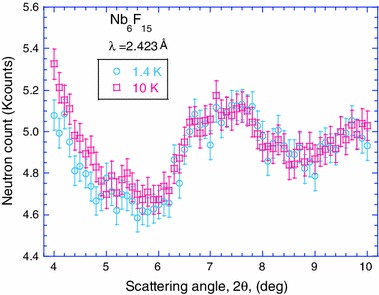
Low-angle neutron powder scattering data at 10 and 1.4 K

On comparing the 1.4 K with the 10 K data, no new reflections were observed in the 1.4 K (i.e., below the magnetic transition temperature) data. Furthermore, a Rietveld analysis of the full neutron profiles (0.18 Å^−1^ ≤ *Q* ≤ 3.47 Å^−1^) did not show any line shifts or line broadening below the magnetic transition. However, for 2θ < 6º, i.e., *Q* < 0.27 Å^−1^, a reduction of the intensity of the low-angle diffuse scattering (Fig. 7) is observed on cooling, that could be ascribed to the removal of paramagnetic spin scattering due to spin ordering. The average fraction of this reduction in the angular range 4^o^ ≤ 2θ ≤ 4.4^o^ is equal to 0.034 (9) and is statistically significant. An estimate of the expected fractional reduction was made using the neutron cross sections for paramagnetic spin (1/2) scattering, incoherent (Nb, F, V) and multiple scattering [19], that yielded 0.041, in agreement with the observed value.

## Discussion

The magnetic properties of the Nb_6_F_15_ compound is a result of the presence of one unpaired electron on the (Nb_6_F_12_^i^)^3+^ cluster core. Its paramagnetic behavior has been established earlier [14]. Owing to the presence of F^a−a^ fluorine bridges between magnetic clusters, it is reasonable to expect at low temperature a transition to an ordered magnetic state. Such a transition to an antiferromagnetic (AF) state is reported in this paper by the combination of several spectroscopic, macroscopic and diffraction techniques. The most direct indications of the AF transition are the significant change of the lineshape of the NMR of the F^i^ but not of the F^a−a^ (Fig. 2).

The inner fluorine F^i^ belongs to the cluster core and senses the hyperfine field that is generated by the single electron in the a_2u_ orbital. The value of this field is estimated, from the width of the NMR spectra at *T* = 3.3 K, to be on the order of 2.5 mT. In typical AF compounds, such as MnF_2_ and CoF_2_ where the unpaired electrons are localized around the magnetic ion, the hyperfine field is on the order of 5–10 T [20, 21]. The very small value of the hyperfine field in the Nb_6_F_15_ can be estimated in the following way. In MnF_2_ the density of the magnetic moment is 0.13 µ_B_/Å^3^. In Nb_6_F_15_, this value is 6 × 10^−4^ µ_B_/Å^3^. This small value results from the large value of the unit cell and the small value of the magnetic moment as was measured in this work.

Although structural changes were not directly observed by the present neutron powder diffraction investigation (no new diffraction peaks and no weak shift or broadening of the diffraction lines), they have been evidenced by ^19^F NMR studies and in particular by the evolution of the F^a−a^ signal versus *T*. Indeed, it has been noted previously [2] that the position of the apical F^a−a^ is temperature-independent for temperature *T* > 75 K. This was attributed to the fact that the F^a−a^ site has cubic symmetry causing cancelation of the magnetic fields that were generated by the neighboring magnetic cluster core. The fact that the signal from the F^a−a^ displays a temperature dependence for *T* < 6 K (at the magnetic transition temperature) may be explained by a deviation from the high-temperature cubic symmetry.

The decrease of the low-angle background upon cooling from 10 to 1.4 K is statistically significant at scattering angle 2θ < 6º, i.e., *Q* < 0.27 Å^−1^. A decrease of that sort is consistent with the emergence of magnetic ordering, which leads to the removal of the paramagnetic scattering contribution to the background. Furthermore, the unusually small angle at which this decrease is observed is consistent with a magnetic form factor of a large magnetic scatterer, namely, a cluster of ions and is a clear signature of the unpaired electron delocalization. The magnetic signal associated with one unpaired electron delocalized on the (Nb_6_F_12_^i^)^3+^ cluster core must be very weak. This feature and the small form factor explain why no supplementary magnetic Bragg peaks were observed below the temperature of magnetic ordering in the conditions used for the neutron diffraction data collection. Our future challenge will be to locate the magnetic spin below the latter temperature by the means of neutron scattering using polarization analysis and/or magnetic field, in particular at low angle.

## Conclusions

It has been shown using NMR, magnetic susceptibility, EMR and neutron powder diffraction, that at temperature below *T*_*N*_ ~5 K the cluster compound Nb_6_F_15_ exhibits a transition to an ordered AF magnetic phase. This ordered phase is a result of interaction between the magnetic (Nb_6_F_12_)^3+^ cluster cores. Cluster magnetism, in this case, is due to a single unpaired electron delocalized over the entire cluster core.
